# Reframing Validity: How Values Shape Assessment in Postgraduate Medical Education

**DOI:** 10.5334/pme.2818

**Published:** 2026-09-16

**Authors:** Sandra Monteiro, Matthew Sibbald, Matthew Lineberry, Teresa M. Chan

**Affiliations:** 1 Department of Medicine, DeGroote School of Medicine, Faculty of Health Sciences, McMaster University, Hamilton, ON, Canada; 2 Hamilton Health Sciences Centre, Hamilton, ON, Canada; 3 Niagara Health Systems, St. Catharines, ON, Canada; 4 Industrial and Organizational Psychology, Rockhurst University, Kansas City, MO, United States; 5 School of Medicine, Toronto Metropolitan University, Brampton, ON, Canada; 6 William Osler Health System, Brampton, ON, Canada; 7 Institute of Health Policy, Management and Evaluation, University of Toronto, Toronto, ON, Canada; 8 Chinese University of Hong Kong, Shenzhen, Shenzhen, China

## Abstract

**Introduction:**

Values (value judgments, beliefs, etc.) have implications for clinician educators’ conceptualization of postgraduate education, as well as their interpretation and application of assessment data. Seeking clarity around individual, program, and systems values within a competency based medical education (CBME) program, the authors sought evidence of distinct values reflected in the CBME assessment literature.

**Methods:**

The authors conducted an exploratory critical review study in two phases aligned with Kahlke’s method. In phase one (from September 2020 to December 2022) the authors worked towards five interconnected goals: focus, generate data, appraise, sample, and analyze. In phase two (October 2022), the authors invited peer review of the process and findings.

**Results:**

Three values and related assessment goals were distilled from the literature on competency-based assessment: 1) Safety: Assessment protects patient safety, ensuring that only clinicians who demonstrate safe practice are advanced; 2) Development: Assessment supports the development of learners’ competence, rather than ranking or differentiating between individuals; and 3) Agility: Assessment evolves in response to context and should be adapted to better support learners and faculty. The authors linked the values within assessment programs to practical consequences for measuring clinical competence.

**Discussion:**

The authors articulated how values within academic medicine directly shape the interpretation and judgement of competence. Deliberately balancing the values of patient safety, learner development, and systems agility can prevent harm when any single value dominates. Preparing faculty to recognize and navigate these value tensions is essential for fair, transparent, and defensible assessment decisions in CBME.

*“Values are intrinsic to the meaning and outcomes of the testing and have always been. As opposed to adding values to validity as an adjunct or supplement, the unified view instead exposes the inherent value aspects of score meaning and outcome to open examination and debate as an integral part of the validation process. This makes explicit what has been latent all along, namely, that validity judgments are value judgments*.” Messick, 1995 [1]

## Introduction

Group-based decisions about trainee progress are now a feature of postgraduate training internationally: clinical competency committees are required in the United States, and training programs in the United Kingdom rely on Annual Review of Competence Progression panels for a similar purpose. What differs across education systems are the content that drive deliberations (e.g. the content in observed tasks or testing), rather than the interpretive work of educators judging competence from imperfect data. In Canada, each postgraduate medical and surgical specialty training program must form a Clinical Competence Committee (CCC) for the purpose of reviewing the progress of medical residents. Based on their review, CCCs can apply remediation plans, recommend transfer to another specialty, or fast track a trainee towards the next stage in training. A tension between standardized and learner-centered assessment goals was noted by the first author, as a non-voting member of two committees.

CCC1 conducted lengthy, policy-driven, standardized reviews of all data in each trainee file, emphasizing patient safety; members treated every data point as evidence requiring verification. Conversely, the Chair of CCC2 synthesized and summarized data so that committee meeting time could focus on trainees needing support, creating a learner-centered process. However, the outcomes of both review processes were similar – trainee progress was deemed appropriate and sufficient in most cases – at most, one trainee required further attention and support and there was very little discussion about trainees who outperformed their peers. It seemed that when patient safety was considered the most important value, CCC1 engaged in a labour-intensive review process that may not have been warranted, and possibly offered little benefit as there was little time left to outline remediation plans. When learner progress was considered the most important value, CCC2 relied on data summaries to identify trainees that may require more support, allowing for rich discussion about remediation plans. It seemed that the value that was prioritized influenced which data was considered most valid for discussion.

Clinician educators tasked with assessing competence, hold different views about what outcomes matter, the purpose of assessment, and what counts as credible, or valid, evidence [1,2,3,4,5,6,7,8,9,10,11,12,13,14,15,16,17]. These different views have been described as contrasting philosophies (i.e. epistemologies) about knowledge as generalizable or context-specific [10,11,12,13,14,15]. Aiming to advance these scholarly conversations, we propose that personal values shape the application of epistemologies, how clinician educators conceptualize, design, and use in-training assessment data—and that making these values explicit strengthens the defensibility of assessment decisions. Examining our values offers greater insight into variations in assessment processes and decisions [7,16]. Decision makers may interpret the same data differently and transparency about those underlying values is essential for coherent and fair assessment practice.

Building on Messick’s assertion that “validity judgments are value judgments [1],” we define values broadly to include values, beliefs, and value judgments. In this paper, we consider values to represent the priorities that shape what matters in assessment (e.g., expertise, compassion). We consider beliefs as personal convictions (e.g. I feel *obligated* to make decisions they can justify to learners, peers, and the public), and value judgments as moral or professional obligations embedded in assessment systems (e.g. Assessment decisions *should* include a clear, transparent rationale). Although these constructs overlap, distinguishing them clarifies how personal reasoning and normative expectations might shape interpretations of assessment data. Throughout this paper, we use *values* to encompass all three, except where distinctions clarify practical examples.

To examine the phenomenon of values-based decision making about clinical competence, observed through a lived experience of the first author, we initiated a theory-focused dialogue grounded in our collective lived experiences [18,19]. Here, we use *theory* in its socio-cultural sense: a set of interpretations that frame how we understand a complex educational process [20]. This lens guided our question for the critical review: What evidence supports our theory that values influence validity evidence in the context of competency based medical education (CBME) and postgraduate assessment?

## Methods

**Figure 1:**
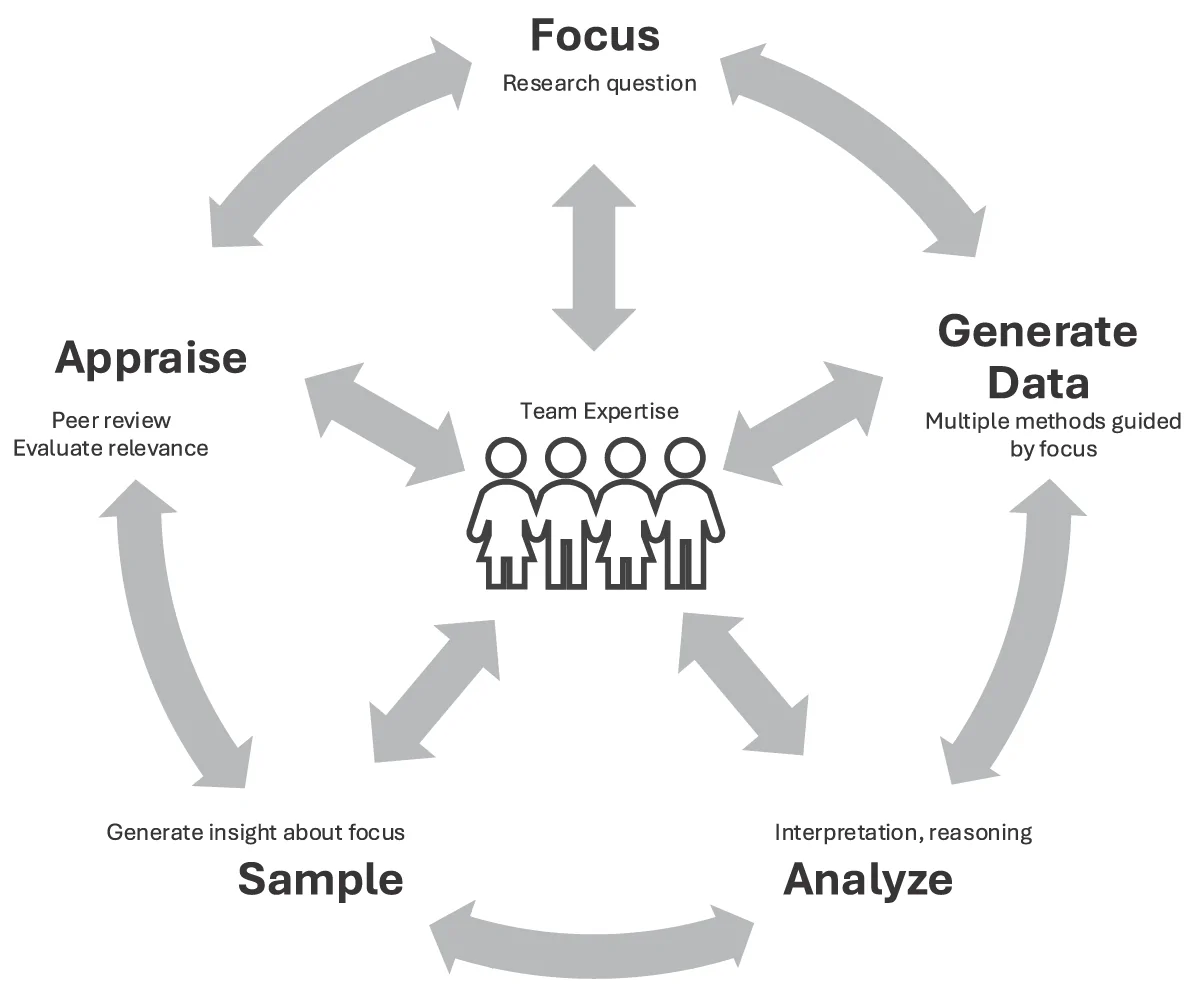
“Elements of a Critical Review” adapted with permission from Kahlke R, Lee M, Eva KW. Building blocks for critical reviews in health professions education. *Journal of Graduate Medical Education*. 2023 Apr 1;15(2):186–9.

We (all four authors) incorporated guidance from Kahlke and colleagues [21,22] to describe this critical review in two phases. In phase one, we worked towards five interconnected goals: focus, generate data, appraise, sample, and analyze. We have provided a graphic representation of these goals in Figure 1. As these goals are highly interactive – neither distinct nor linear – we have emphasized (i.e. bolded) each within our methods below. In phase two, we invited peer review of our process and findings from phase one.

While procedures for empirical studies are typically a set of sequential distinct steps, a critical review offers flexibility, permitting the sharing of preliminary findings within the methods, as these influence the goals [21,22]. In the following passages we first share our team reflexivity, and then describe an iterative multi-phase, multi-method critical review procedure, which blends objective formation, data generation, analysis, and preliminary findings.

### Team Reflexivity

Given the exploratory nature of this review, we do not claim to account for all possible interpretations. Rather, we aim to spark conversation about the diversity of values that shape postgraduate assessment. We offer this account to clarify our influence on the methods and results. Our team comprises four health professions education researchers—two experienced clinician educators with multiple degrees in education and two PhD-trained psychologists—whose disciplinary diversity shaped both our questions and interpretations. Together, we bring expertise in clinical practice, simulation, cognitive and organizational psychology, CBME, and program leadership, and we share a commitment to evidence-informed and socially accountable scholarship grounded in a social constructivist view of knowledge – that is we believe that *truth* is multifaceted and many *truths* can exist within the same situation.

Our breadth of expertise allowed us to challenge assumptions, surface implicit beliefs, and triangulate perspectives as we examined how values influence judgments of validity. The clinician authors contributed insights from program leadership and competence committee facilitation. The non-clinician authors, trained in cognitive and industrial-organizational psychology, brought experience in decision science, assessment design, and epistemology; one has served on two competence committees, and both contribute nationally and internationally to assessment scholarship. The first author also drew on prior experience directing a competency-based assessment design centre and advising health professions regulatory administrators across Canada.

We recognize that our epistemologies—shaped by work in clinical reasoning, cognitive science, organizational behavior, data analytics, and systems thinking—inform how we frame assessment problems and what we consider credible evidence. We invite readers to consider how their own values influence their judgments of assessment validity. Our beliefs about validity, reliability, trust, and fairness are intertwined with our professional identities. Through interdisciplinary dialogue, we developed a shared theoretical stance central to our research questions: individuals draw on context-specific values when interpreting assessment data, and these values shift across situations and over time. We began our discussion about values and validity in 2020 and present our original findings based on the critical review methodology described below, which concluded in 2022. All assessment scholarship cited within the Methods and Results sections were accessible at that time; published online before 2023. We then conducted a focused update of the literature (January 2023 to June 2026) to identify scholarship published after our original search concluded; these sources are integrated into the Discussion to situate our findings within the current conversation.

### Critical Review Procedure – Phase 1

To establish a focus for our discussions, we sampled and appraised peer reviewed medical education literature (i.e. 2000 to 2026 since this was the period within which CBME became prominent) for guidance, taking inspiration to derive our objectives from a limited set of publications that aligned with our research objectives: St-Onge and colleagues [6,23,24] and Cook & Lineberry (2016) [25]. The work of these scholars (St. Onge and colleagues; Cook & Lineberry) introduced sensitizing concepts, such as scholarly beliefs framing validity as both a “social imperative” and an “argument-based evidentiary chain” [6,23,24,25,26], which later influenced what we analyzed from transcripts of our discussions. Across several months of meetings, our recorded discussions, reflective notes, and key articles helped us surface various beliefs shaping assessment discourse. Our first elaboration of Messick’s statement (i.e. “*validity judgments are value judgments”*) was that shifting value orientations create methodological challenges—selecting from the multitude of methods to generate, represent, and interpret data —and complexity in assessment. We then directed our attention toward identifying the value judgments most visible in the literature [27,28,29,30,31,32].

In an iterative process, we continued to sample and appraise the literature that were cued by our discussions and through reference searching of discussed papers, retaining empirical studies that addressed our research questions (please see appendix in Supplementary Material 1 for the final list of sources), while returning to analyze transcripts. We documented how beliefs rooted in psychometric traditions influenced interpretations of workplace-based assessment data. Written comments, EPA scores, and verbal attestations were frequently viewed through a quantitative lens, reinforcing assumptions about reliability and objectivity [33,34,35,36,37,38,39,40]. Scholars highlighted the burden on faculty tasked with adapting curricula to align with CBME frameworks [32,41,42,43], others examined tensions between patient-safety priorities and learner-centered assessment practices [8,44,45,46].

At this point we proposed a second elaboration of Messick’s statement, noting that value judgments most visible in the assessment literature were patient safety, learner development, assessment traditions, community, trust, and quality [7,16]. Conversely, the literature lacked sufficient content about talent [47,48]. In our team discussions, we noted that talent was a strikingly absent value in both teaching and assessment practices, despite its importance to long-term workforce development [47]. Few papers explicitly addressed the intersection of talent and performance, even though talent is often selected for and valued [48].

We distilled these preliminary findings into 5 statements. These value judgments are presented in Table 1, paired with representative quotes from our team discussions, and 7 overarching values.

**Table 1:** Five recurrent value judgments observed in postgraduate medical education assessment discussions, supported by representative quotes from meeting transcripts and analytic memos. These value judgments—patient safety, learner development, tradition, talent cultivation, and trust in community—illustrate how underlying values shape interpretations of assessment data and contribute to differing perceptions of validity.


VALUES	VALUE JUDGMENTS	QUOTES AND RATIONALE

Patient Safety	It is critical to measure trainee performance to determine their fitness for practice, to ensure patient safety	“To kick somebody out of the program. I have to generate reliable evidence… they have irremediable performance gaps.” “Data that are not reliable should not be trusted to predict future competence… it is unethical to use unreliable data to support decisions that impact which trainees advance.” “For CBME, the goal is only to ensure identification of individuals who are not safe.”

Learner Development	Trainee improvement and learning outcomes are the core purpose of health professions education and medical education. (appears throughout transcripts where remediation and coaching are described)	“We just say that we would like to get you more time to learn this because you seem to need more time to be able to achieve this.” “We have shifted away from measurement of pass/fail to being about continued development…” “The goal… is to understand a complex situation with the hope that moves the resident forward.”

Tradition	Assessment is a cultural tradition that is important to uphold.	“There is a history and a tradition that we are writing within, so we just have to acknowledge that.” “It serves big testing companies… it is designed for those folks… there have been … pressures to [incorporate] psychometrics that exist right now.” “That is the more traditional way of thinking about how we have been doing assessment in the past… mastery at the end.”

Talent	Talent should be fostered and developed within learners to allow for maximal impact.	“We actually have not ever told somebody they are not ever going to be able to learn this… we would like to get you more time to learn this.” “Some people can practice in a lot of environments… some people have really narrow practice environments… they have to choose wisely.” “The act of assessment… is used as the starting point to understand a complex situation with the hope that moves the resident forward.”

Community	Assessment systems should include and acknowledge the importance of faculty members. Faculty members are essential to accomplish the goals of assessing trainees.	“We don’t have good performance data on faculty… sometimes the problem is the faculty member, not the resident.” “Bigger concerns arise when raters know themselves to be unreliable or invalid…” “Unless you have very tight circles of people that are trained and savvy to interpret those signals… things fall apart.”


### Consultation Process – Phase 2

We invited several non-author consultants to provide a peer review of the content in Table 1. Instead of including quotes we sent consultants a brief video of the first and last author describing our team’s perspective. Six consultants were nominated by the author team and selected for their expertise in health professions education and assessment; all agreed to participate. Among them were people that held a MD/PhD, or PhD. All were health professions education scientists with varied expertise in quantitative and qualitative research methodology. Two consultants had specific expertise in critical reviews, two in competency-based assessment, and one provided additional insights as a clinician educator. Four lived in Canada, and two in the United States of America. Each consultant was invited to provide feedback via a brief survey which summarized the goal of the paper, the key phenomenon, our theory, proposed value judgments, and several assumptions related to the role of values in assessment. This consultation process was submitted to the Hamilton Integrated Research Ethics Board (HiREB), which granted an exemption confirming that the activity did not constitute human subjects research requiring ethics review.

#### Consultant Feedback

The survey questions are available in the appendix (Supplementary Material 2). From the 5 options, all consultants endorsed Patient Safety and Learner Development. The statement about Community was second, receiving support from 4 consultants. Some consultants emphasized the purpose of assessment to serve as gatekeepers and uphold public trust. While others reinforced our goal of making personal values explicit.

We integrated this feedback with our previous findings to identify 3 values: (Patient) Safety, (Learner) Development and (Systems) Agility. Targeting literature from our search that promoted, or problematized overinvestment in these value judgments, we returned to our discussions asking these sub-questions:

- *What personal beliefs arise from these value judgments?*
- *What are some practical implications of the relationship between these values and validity?*

We have organized answers to our sub-questions in Table 2, stating a value, value judgment, and belief, as well as potential opportunities for initiating a discussion about values and validity.

**Table 2:** Values, value judgments, beliefs, and risks associated with each, to illustrate how core beliefs can shape faculty decisions, and highlight risks that emerge when any single value is emphasized. For each of the 3 values, we have provided an example of a belief statement, potential implications of this belief for education programs, potential risks or consequences, and examples of how the value and belief might be operationalized in the behaviour or decision making of faculty. Observing the behaviours described in the last column creates opportunities to initiate conversations about individual faculty assessment values and beliefs.


VALUE JUDGMENTS WE DERIVED FROM ASSESSMENT SCHOLARSHIP	ASSOCIATED BELIEFS & IMPLICATIONS FOR EDUCATION PROGRAMS	RISKS & CONSEQUENCES	OPPORTUNITIES TO DISCUSS BROADER PROGRAM VALUES

Safety: Assessment protects patient safety above all else, ensuring that only clinicians who demonstrate safe practice are advanced.	We believe that assessment influences patient safety. Therefore, programs must shape who enters and progresses within the profession.	Prioritizing this belief can justify overly conservative, exclusionary assessment practices that prioritize avoidance of risk over supporting learning—potentially reducing workforce diversity and worsening shortages. Systemic racism and other forms of discrimination are exacerbated by these priorities.	Faculty raise safety red flags, calling for immediate dismissal of trainees, without attending to process. Faculty fail a learner or delay progression whenever they feel unsure, guided by suspicion rather than fair process. Faculty gravitate toward structured checkboxes to minimize the chance of “missing something,” resulting in narrow assessments that overlook growth, context, or accessibility needs.

Development: Assessment supports the development of learners’ competence, rather than ranking or differentiating between individuals.	We believe that learners develop competence through meaningful assessment experiences that inform their growth. Therefore, programs are responsible for learners’ success.	Prioritizing this belief may underplay the importance of accountability, allowing significant performance concerns to go unaddressed in the pursuit of promoting growth. These decisions can mask imminent or future risks to patients or teams, inadvertently preventing early identification of patterns that require intervention. Conversely, learners with accessibility needs can be overlooked or excluded.	Faculty hesitate to document or discuss critical feedback to avoid discouraging the learner. Faculty give higher ratings than warranted to maintain a supportive environment.

Agility: Assessment evolves in response to context and should be adapted to better support learners and faculty in health professions education.	We believe that assessment practices reflect the contexts in which they are used. Therefore, programs must adapt as those contexts evolve.	Prioritizing this belief may lead to constant revisions of assessment tools and processes, increasing faculty workload and creating ongoing demands that are difficult to sustain alongside clinical and teaching responsibilities. Consequently, increased faculty workload will further strain healthcare services.	Faculty repeatedly update assessment tools in response to each new context, rotation need, or curricular concern, creating an unending cycle of redesign that adds to administrative burden. Faculty tailor processes for each learner or setting, valuing contextualization but unintentionally generating inconsistent expectations and additional work to manage one-off solutions.


## Results

A total of 67 papers were reviewed and analyzed by our authorship group. In answer to our central question, we found ample evidence (i.e. empirical studies) to support our theory (Supplementary Material 1). Here we describe the evidence supporting the 3 values.

### Safety: Assessment protects patient safety above all else, ensuring that only clinicians who demonstrate safe practice are advanced

Safety was a dominant value in the literature, particularly in work from the early 2000s that framed assessment as essential for ensuring readiness for unsupervised practice. This focus often led to calls for psychometrically sound, objective assessments and correlation studies intended to validate the necessity of formal evaluation systems [35,36,37]. Several papers promoted investment in data infrastructure and predictive analytics to identify unsafe performance, while others cautioned that variability in clinical teams and settings limited the reliability of such approaches [38,39,40]. This contextual sensitivity highlighted the vulnerability of standardized assessments to small changes in sampling, which can distort score accuracy [27]. There was also growing interest in linking patient-care metrics with learning analytics to strengthen outcome measurement in CBME, reinforced by broader trends in clinical informatics [28,29,30]. If safety is prioritized as the only value, we may compromise growth and agility in favour of strict criteria, defensibility, and threshold-based judgments—despite evidence that the predictive validity of large scale standardized assessments, while positive and typically statistically significant, is quite small in relation to the total variance in measures of patient safety indicators [36,37,38,39,49,50,51,52].

### Development: Assessment supports the development of learners’ competence, rather than ranking or differentiating between individuals

A second dominant value in the literature was learner development, reflected in the increasing prominence of assessment for learning [53,54,55], such as coaching and mentorship models: R2C2, surgical coaching, and the Calgary Audit-and-Feedback model [31,56,57,58,59,60]. This body of work positions trainee development as the central purpose of health professions education and highlights the need for assessment systems that are fair, useful, and developmentally oriented [61]. In this context, competency frameworks from regulators and specialty disciplines serve to clarify expectations for trainees and the public, rather than to provide strict criteria. Literature that values development, focus on supporting completion of training rather than discriminating between trainees. Investing in learner development means identifying learning needs, providing coaching and skills training, and preparing trainees for key assessments. Recent discussions of assessment for and as learning reinforce the importance of thoughtful data curation and fairness as foundations for learner-centered outcomes [61,62]. If development is prioritized as the only value, we may minimize safety concerns, or only focus on struggling learners, which may devalue excellence.

### Agility: Assessment evolves in response to context and should be adapted to better support learners and faculty in health professions education

A third value reflected in the literature was agility—the expectation that assessment systems should evolve with changing educational, clinical, and societal contexts. Medical education, more so than other health professions, has long relied on traditional models emphasizing hierarchy, summative judgment, and high-stakes performance—often functioning as rites of passage. These paradigms persist even as CBME promotes more flexible, developmental approaches.

Valuing agility requires innovation and an explicit effort to disrupt outdated assessment rituals that can undermine learning cultures. Studies highlighted how entrenched traditions may produce shame, foster fear of failure, and make trainees hesitant to learn openly—even in feedback-rich settings where faculty double as assessors [45,63,64,65,66,67,68,69,70,71,72,73]. Assessment systems must evolve to meet the needs of society, and decrease the burden of assessment so that learners and faculty can stay focused on patient care [74]. High-stakes examinations were frequently cited as barriers to equity in selection and advancement, particularly for underrepresented groups. Innovations in assessment practices are critical to help ensure representation and inclusivity in healthcare. In times of resource constraints, such as during the peak of the COVID-19 pandemic, many trainees (e.g. medical and nursing students, medical residents) were trusted with advanced responsibilities, playing a critical role in sustaining healthcare [75,76]. Healthcare organizational resources remain constrained and strained worldwide, signaling a critical need to adapt and formally consider “accelerated graduation” processes [75,76]. If agility is prioritized as the only value, in the absence of additional resources, we may create unsustainable demands for faculty to engage in constant quality improvements of teaching and assessment tools.

## Discussion

A wide range of human values may be implicated in assessment, but what matters is which become dominant and how they shape decision-making. Comparable insights appear in other fields: for example, Birhane et al. identified more than 60 values in highly cited machine learning literature, with justice and fairness rarely emphasized and performance prioritized in nearly all cases [77]. Our findings similarly challenge assumptions that postgraduate assessment is value neutral. When safety is prioritized above all else, strict, objective, and normative processes tend to dominate.

In this critical review, we sought evidence to support the proposed relationship between values and validity, and the influence of values on clinician educators’ conceptualization, design, and use of in-training assessment data. Synthesizing the evidence derived from assessment theories, our expertise, lived experiences and peer reviewed literature, we identified three recurring values—safety, development, and agility—that underpin much of the recent literature on assessment. By moving beyond analyses of the merits of assessment designs (e.g., standardized vs. workplace-based assessments) [11,33] and their links to different philosophies [11,12], we show how unspoken values—an aspect of context-specificity [7,16,78,79,80,81]—shape the interpretive work of clinician educators. Although previous work has linked values to rater variability and subjectivity [10,11,12,82,83], few studies have examined how broader, context-specific values influence high-stakes decisions and perceptions of validity in postgraduate assessment [7,81,84,85,86].

Safety-oriented literature emphasized rigorous data infrastructure, standardized measures, and the linkage of learning analytics to patient outcomes [27,28,29,30,34,35,37,38,39,40,49]. Early dominance of the psychometric era meant that safety, fairness, acceptability, and defensibility were often treated as implicit outcomes of numerical reliability and validity. Development-oriented literature emphasized coaching and mentorship models [31,57,58,87,88]. Work emphasizing systems agility critiqued the persistent influence of traditional, high-stakes assessment rituals—rites of passage that can promote shame, fear of failure, and inequities—and called for systems that evolve to better support learners and faculty [45,63,64,65,66,67,68,69,70,71,72,73].

Scholarship published since our search closed has largely moved toward the position we advance here. *Perspectives on Medical Education*’s “Next Era in Assessment” collection calls for assessment that is development-oriented and socially accountable [89], and names trust—individual, organizational, and societal—as a foundational design value for assessment systems [90]. Interviews with validity experts describe validation as rhetorical work, built to persuade particular audiences and therefore contingent on their values [91], and analyses of standard setting trace how pass/fail decisions shift with prevailing epistemic regimes rather than resting on measurement fact alone [92]. Studies of competency committees document members filtering the same data through local standards and personal reasoning [93], while a narrative review concluded that the downstream impact of these committees on trainees and patient care remains largely uninvestigated [94]. The developmental and agility threads have also matured: programmatic assessment has been formalized as assessment for learning at the level of an AMEE Guide [95], new work refines the mechanisms of R2C2 coaching conversations [96], and Canadian studies describe both the burden created by variable assessment forms [97] and the principled adaptations educators make to reconcile system design with local realities [98]. Parallel reviews of bias in observed assessment, and of efforts to mitigate it, position fairness as both a threat to validity and a value in its own right [99,100], and the emerging precision education movement adds personalization and efficiency to the list of values warranting the same scrutiny [101].

While safety, development, and agility appear prominently in recent scholarship, talent rarely surfaced as an explicit value guiding assessment design or educational practice. This silence is meaningful: it reflects a legacy in which assessment systems treated talent as normative, rather than acknowledge diverse capabilities of individuals who may contribute differently—and substantially—to the workforce [48]. It is also an opportunity for the field to question its long-standing assumptions about the purpose of evaluation and to consider how systems might better acknowledge learners not only as meeting thresholds of competence but also as emerging clinicians with strengths, potential, and future contributions. A recent scoping review substantiates this gap: of 189 studies engaging with talent in medical education, only a quarter used the term explicitly and just seven offered a definition, with most framing talent as something to be identified or sorted rather than developed [102]. Read this way, the absence is a diagnostic rather than an oversight: assessment systems built to sort learners against thresholds have little vocabulary for strengths, so committee attention pools around struggling trainees while distinctive capability goes unremarked. However, if we are seeking to further value our human capital within healthcare, then perhaps competence committees could be asked to attend to both those who struggle (e.g. identify need for remediation) and for those who show unusually quick progression (e.g. fast track or enrich a training experience for a learner). For scholarship, it points to selection research and strengths-based assessment design as places where a developmental account of talent could be built and tested.

Different values can drive data driven decision making to gain new insights from various vantage points [103,104,105] about trainee progress tracking [106], faculty development [107], trainee competency [108], and curricular change [107]. In non-standardized clinical in-training settings where assessment data are often incomplete [109,110,111], or qualitative in nature, attention to data reliability is inappropriate and potentially unethical [40,112]. Mathematically, reliability places an upper limit on validity [32,33,34,113,114], yet this relationship is only meaningful under certain conditions. For example, the predictive promise of learning curves makes them useful [29,32,115,116,117] if the data are sufficient and reliable, conditions that are rarely met in some contexts (e.g. Canada [80,118,119], where EPAs are aligned with terminal stages of clinical competence, rather than across all of postgraduate training). In this context, pursuing the construction of learning curves in spite of this may waste time and resources [32,120].

We urge decision makers and scholars in CBME to begin a more nuanced discussion around the wide and varied purposes for assessment data. When decision makers declare and discuss their values within systems, they make clear what they expect from the data. Stated expectations can reveal the path to reflexive and robust data analysis for the intended purpose [43]. Through discussions of values, decision makers may then realize that the same data, when reviewed and seen by others with different values can yield different insights. This requires a fundamental shift in data usage and governance in CBME beyond values steeped in experimental and positivist perspectives. The relative weight these values carry will differ across regulatory and cultural contexts—systems vary in how much discretion committees hold and in the maturity of their CBME implementations—so we offer this framework as a lens for local conversations rather than a prescription drawn from any single system.

## Limitations

We brought together experienced educational practitioners and scholars to examine how values shape judgments of assessment validity. Our goal was to spark a conversation relevant across professions and settings; however, we do not claim that our emerging theory generalizes to all assessment decisions or contexts. Because this work aimed to build theory rather than catalogue every possible value, we focused on the most prominent values reflected in recent postgraduate assessment literature. Other values undoubtedly influence assessment—such as fairness, credibility, fitness for purpose, transparency, and defensibility—and the relative absence of scholarship on “talent” highlights how some values remain underexplored [5,7,16,48,85].

## Conclusion

Assessment decisions are filled with tension, in part because stakeholders often work from different assumptions and expectations. Assessment complexity can be reduced by foregrounding the broad values of safety, development and agility, which likely shift by context, even for the same clinician educator.

As assessment systems expand across diverse contexts in CBME, these differences become more visible—and more consequential. How assessment information is interpreted, and how it informs decisions, ultimately depends on the values educators bring to their judgments. Making those values explicit is essential for coherent, fair, and defensible assessment practice.

## Additional Files

The additional files for this article can be found as follows:

10.5334/pme.2818.s1 Supplementary Material 1. In phase 2, this survey was presented to all consultants asked to review the phase 1 findings of this critical review.

10.5334/pme.2818.s2 Supplementary Material 2. The following are the literature that were reviewed and appraised by the co-authors of Monteiro et al. Reframing Validity: How Values Shape Assessment in Postgraduate Medical Education. The relevant literature are organized by the three values of safety, development and agility.
