# Supplementary material for: Reframing Validity: How Values Shape Assessment in Postgraduate Medical Education
Source: Perspect Med Educ. 2026 Sep 16;15(1):830–44. doi: 10.5334/pme.2818 (PMC13588219; doi:10.5334/pme.2818)
Supplement: Supplementary file 1 [file pme-15-1-2818-s1.pdf]

Supplemental Materials 1

The following are the survey questions that were asked of the consulting experts by the co-authors of Monteiro et al. Reframing Validity: How Values Shape Assessment in Postgraduate Medical Education.

1. Which of the value statements do you feel is reflected in the HPE or MedEd literature around assessment?
    a. Patient Safety - It is critical to measure trainee performance to determine their fitness for practice, to ensure patient safety
    b. Learner Development - Trainee improvement and learning outcomes are the core purpose of health professions education and medical education. (appears throughout transcripts where remediation and coaching are described)
    c. Tradition - Assessment is a cultural tradition that is important to uphold.
    d. Talent - Talent should be fostered and developed within learners to allow for maximal impact.
    e. Community - Assessment systems should include and acknowledge the importance of faculty members. Faculty members are essential to accomplish the goals of assessing trainees.
2. Are there any statements that you feel are missing?
3. What do you think about this value-driven analysis of the literature?
4. What aspects do you like about this proposed framing based on values?
5. What are your concerns about this proposed way of examining the literature? Is there any problem with developing or using a value-statement-driven approach towards an assessment system?
6. What are some advantages of thinking about the HPE literature in this way?
7. How do you see your own writing reflected by one or multiple of these value statements? (If so, which one(s)?
