# Supplementary material for: Reframing Validity: How Values Shape Assessment in Postgraduate Medical Education
Source: Perspect Med Educ. 2026 Sep 16;15(1):830–44. doi: 10.5334/pme.2818 (PMC13588219; doi:10.5334/pme.2818)
Supplement: Supplementary file 2 [file pme-15-1-2818-s2.pdf]

Supplemental Material 2:
The following are the literature that were reviewed and appraised by the co-authors of Monteiro et al. Reframing Validity: How Values Shape Assessment in Postgraduate Medical Education.

Safety

Reference	Patient Safety Focused Theme
Kogan JR, Conforti LN, Iobst WF, Holmboe ES. Reconceptualizing variable rater assessments as both an educational and clinical care problem. Academic Medicine. 2014 May 1;89(5):721-7.	Protecting patients through defensible judgments
Kogan JR, Holmboe ES, Hauer KE. Tools for direct observation and assessment of clinical skills of medical trainees: a systematic review. Jama. 2009 Sep 23;302(12):1316-26.	
Ten Cate O, Hart D, Ankel F, Busari J, Englander R, Glasgow N, Holmboe E, Iobst W, Lovell E, Snell LS, Touchie C. Entrustment decision making in clinical training. Academic Medicine. 2016 Feb 1;91(2):191-8.	
Schuwirth LW, van der Vleuten CP. Programmatic assessment and Kane's validity perspective. Medical education. 2012 Jan;46(1):38-48.	
Holmboe ES. Realizing the promise of competency-based medical education. Academic Medicine. 2015 Apr 1;90(4):411-3.	
Ten Cate O. Medical education from a succession perspective: what can the apprenticeship model bring to competency-based education?. Academic Medicine. 2026 Mar 24:wvag071.	
	Linking assessment outcomes to real-world patient safety
Wenghofer E, Klass D, Abrahamowicz M, Dauphinee D, Jacques A, Smee S, Blackmore D, Winslade N, Reidel K, Bartman I, Tamblyn R. Doctor scores on national qualifying examinations predict quality of care in future practice. Medical education. 2009 Dec;43(12):1166-73	
Tamblyn R, Abrahamowicz M, Dauphinee WD, Hanley JA, Norcini J, Girard N, Grand'Maison P, Brailovsky C. Association between licensure examination scores and practice in primary care. JAMA. 2002 Dec 18;288(23):3019-26.	
Tamblyn R, Abrahamowicz M, Dauphinee D, Wenghofer E, Jacques A, Klass D, Smee S, Blackmore D, Winslade N, Girard N, Du Berger R. Physician scores on a national clinical skills examination as predictors of complaints to medical regulatory authorities. JAMA. 2007 Sep 5;298(9):993-1001.	

Norman GR, Wenghofer E, Klass D. Predicting doctor performance outcomes of curriculum interventions: problem-based learning and continuing competence. Medical education. 2008 Aug;42(8):794-9.	
Tang B, Lam AC, Wankiewicz M, Ismail MF, Roberts SB, Liu C, Schumacher D, Kinnear B, Park YS, Tess A, Verma AA. Developing Resident-Sensitive Quality Measures for Internal Medicine. JAMA Network Open. 2026 May 8;9(5):e2611700.	
Grace, Elizabeth S. MD; Wenghofer, Elizabeth F. PhD; Korinek, Elizabeth J. MPH Predictors of Physician Performance on Competence Assessment, Academic Medicine: June 2014 - Volume 89 - Issue 6 - p 912-919 doi: 10.1097/ACM.0000000000000248	Evaluating programs by the risks their graduates pose to patients
Asch DA, Nicholson S, Srinivas S, Herrin J, Epstein AJ. Evaluating obstetrical residency programs using patient outcomes. JAMA. 2009 Sep 23;302(12):1277-83.	
Epstein AJ, Srinivas SK, Nicholson S, Herrin J, Asch DA. Association between physicians' experience after training and maternal obstetrical outcomes: cohort study. Bmj. 2013 Mar 28;346.	
Chan T, Sebok-Syer S, Thoma B, Wise A, Sherbino J, Pusic M. Learning analytics in medical education assessment: the past, the present, and the future. AEM Education and Training. 2018 Apr;2(2):178-87.	Seeking measurable signals that distinguish safe from unsafe future practice
Ealing IV, Whereat S, Forsyth R, Hong J, Laurence J. Surgeon and trainee perspectives on intraoperative education: a systematic review and meta-synthesis. Medical Education Online. 2025 Dec 31;30(1):2560628.	
Jenkins PD, Willis S, Doberne J, Jesneck J, Thanawala R. EHR-Driven Delivery of EPA Assessments Avoids Cherry-Picking Bias. Journal of Surgical Education. 2026 Sep 1;83(9):104015.	
Ryan MS, Teunissen P, Santen SA, Emke A, Jones RL, Poeppelman R, Kelleher M, Parsons A, Jolani S, Vinson A. "It's about better doctors": exploring the purpose, structure, and function of competency committees in medical school. Academic Medicine. 2026 Feb;101(2):188-97.	
Sebok-Syer SS, Chahine S, Watling CJ, Goldszmidt M, Cristancho S, Lingard L. Considering the interdependence of clinical performance: implications for assessment and entrustment. Medical Education. 2018 Sep;52(9):970-80.	Revealing hidden system factors that distort competence judgments
Thoma B, Warm E, Hamstra SJ, Cavalcanti R, Pusic M, Shaw T, Verma A,	

Frank JR, Hauer KE. Next steps in the implementation of learning analytics in medical education: consensus from an international cohort of medical educators. Journal of Graduate Medical Education. 2020 Jun;12(3):303-11.	
Thoma B, Ellaway RH, Chan TM. From Utopia through Dystopia: charting a course for learning analytics in competency-based medical education. Academic Medicine. 2021 Jul 1;96(7S):S89-95	
Yeates P, Sebok-Syer SS. Hawks, Doves and Rasch decisions: Understanding the influence of different cycles of an OSCE on students' scores using Many Facet Rasch Modeling. Medical Teacher. 2017 Jan 2;39(1):92-9.	

Development

**Reference**	**Learner Development Focused Theme**
Ginsburg S, van der Vleuten CP, Eva KW, Lingard L. Cracking the code: residents' interpretations of written assessment comments. Medical Education. 2017 Apr;51(4):401-10.	Helping learners understand what to do next
Ramani S, Könings KD, Ginsburg S, van der Vleuten CP. Feedback redefined: principles and practice. Journal of general internal medicine. 2019 May;34(5):744-9.	
Watling CJ, Ginsburg S. Assessment, feedback and the alchemy of learning. Medical education. 2019 Jan;53(1):76-85.	
Richardson D, Kinnear B, Hauer KE, Turner TL, Warm EJ, Hall AK, Ross S, Thoma B, Van Melle E, ICBME Collaborators. Growth mindset in competency-based medical education. Medical Teacher. 2021 Jul 3;43(7):751-7.	
Chan TM, Sebok-Syer SS, Sampson C, Monteiro S. The Quality of Assessment of Learning (Qual) score: validity evidence for a scoring system aimed at rating short, workplace-based comments on trainee performance. Teaching and Learning in Medicine. 2020 May 26;32(3):319-29.	
Kalun P, Braund H, McGuire N, McEwen L, Mann S, Trier J, Schultz K, Curtis R, McGuire A, Pereira I, Dagnone D. Was it all worth it? A graduating resident perspective on CBME. Medical Teacher. 2025 Mar 4;47(3):467-75.	
McConnell M, Sherbino J, Chan TM. Mind the gap: the prospects of missing data. Journal of Graduate Medical Education. 2016 Dec;8(5):708-12.	Data entry is an act of educational care—without it, feedback cannot happen
Ramani S, Könings KD, Ginsburg S, van der Vleuten CP. Twelve tips to promote a feedback culture with a growth mind-set: Swinging the feedback pendulum from recipes to relationships. Medical teacher. 2019 Jun 3;41(6):625-31.	
Schuwirth LW, Southgate L, Page GG, Paget NS, Lescop JM, Lew SR, Wade WB, Barón-Maldonado M. When enough is enough: a conceptual basis for fair and defensible practice performance assessment. Medical education. 2002 Oct;36(10):925-30.	Understanding fairness as essential to developmental progress, not separate from it
Valentine N, Durning S, Shanahan EM, Schuwirth L. Fairness in human judgment in assessment: A hermeneutic literature review and conceptual framework. Advances in Health Sciences Education. 2021 May;26(2):713-38.	
Valentine N, Durning SJ, Shanahan EM, van der Vleuten C, Schuwirth L. The pursuit of fairness in assessment: Looking beyond the objective. Medical Teacher. 2022 Jan 28:1-7.	

Colbert CY, French JC, Herring ME, Dannefer EF. Fairness: the hidden challenge for competency-based postgraduate medical education programs. Perspectives on Medical Education. 2017 Oct;6(5):347-55.	
Hauer KE, Edgar L, Hogan SO, Kinnear B, Warm E. The Science of Effective Group Process: Lessons for Clinical Competency Committees. Journal of Graduate Medical Education. 2021 Apr;13(2s):59-64.	
Chan TM, Sherbino J, Mercuri M. Nuance and noise: lessons learned from longitudinal aggregated assessment data. Journal of Graduate Medical Education. 2017 Dec;9(6):724-9.	Turning assessment into a tool for guiding practice, not just judging outcomes
Howard NM, Cook DA, Hatala R, Pusic MV. Learning curves in health professions education simulation research: a systematic review. Simulation in Healthcare. 2021 Apr 1;16(2):128-35.	
Hatala R, Gutman J, Lineberry M, Triola M, Pusic M. How well is each learner learning? Validity investigation of a learning curve-based assessment approach for ECG interpretation. Advances in Health Sciences Education. 2019 Mar;24(1):45-63.	
Pusic M, Pecaric M, Boutis K. How much practice is enough? Using learning curves to assess the deliberate practice of radiograph interpretation. Academic Medicine. 2011 Jun 1;86(6):731-6.	
Pusic MV, Andrews JS, Kessler DO, Teng DC, Pecaric MR, Ruzal-Shapiro C, Boutis K. Prevalence of abnormal cases in an image bank affects the learning of radiograph interpretation. Medical education. 2012 Mar;46(3):289-98.	
Pusic MV, Boutis K, Hatala R, Cook DA. Learning curves in health professions education. Academic Medicine. 2015 Aug 1;90(8):1034-42.	
Carraccio C, Englander R, Holmboe ES, Kogan JR. Driving care quality: aligning trainee assessment and supervision through practical application of entrustable professional activities, competencies, and milestones. Academic Medicine. 2016 Feb 1;91(2):199-203.	Supporting progressive competence through structured, intentional educational design
Carraccio C, Englander R, Gilhooly J, Mink R, Hofkosh D, Barone MA, Holmboe ES. Building a framework of entrustable professional activities, supported by competencies and milestones, to bridge the educational continuum. Academic Medicine. 2017 Mar 1;92(3):324-30.	
Sherbino J, Bandiera G, Doyle K, Frank JR, Holroyd BR, Jones G, Norum J, Snider C, Magee K. The competency-based medical education evolution of Canadian emergency medicine specialist training. Canadian Journal of Emergency Medicine. 2020 Jan;22(1):95-102.	

Sherbino J, Bandiera G, Frank JR. Assessing competence in emergency medicine trainees: an overview of effective methodologies. Canadian Journal of Emergency Medicine. 2008 Jul;10(4):365-71.	
Ten Cate O. Medical education from a succession perspective: what can the apprenticeship model bring to competency-based education?. Academic Medicine. 2026 Mar 24:wvag071.	

Agility

**Reference**	**Systems Agility Focused Theme**
Thoma B, Monteiro S, Pardhan A, Waters H, Chan T. Replacing high-stakes summative examinations with graduated medical licensure in Canada. CMAJ. 2022 Feb 7;194(5):E168-70.	Evolving assessment systems to better fit the realities of modern training.
Acai A, Li SA, Sherbino J, Chan TM. Attending Emergency Physicians' Perceptions of a Programmatic Workplace-Based Assessment System: The McMaster Modular Assessment Program (McMAP). Teach Learn Med. 2019;31(4):434-444. doi:10.1080/10401334.2019.1574581	
Li S, Sherbino J, Chan TM. McMaster Modular Assessment Program (McMAP) through the years: Residents' experience with an evolving feedback culture over a 3-year period. AEM Educ Train. 2017;1(1):5-14. doi:10.1002/AET2.10009	
Holland C. Critical review: medical students' motivation after failure. Adv Health Sci Educ. 2016;21(3):695-710. doi:10.1007/s10459-015-9643-8	Adapting educational systems to reduce harm and support healthier responses to error and struggle
Kandasamy S, Vanstone M, Colvin E, Chan T, Sherbino J, Monteiro S. "I made a mistake!": a narrative analysis of experienced physicians' stories of preventable error. Journal of Evaluation in Clinical Practice. 2021 Apr;27(2):236-45.	
Bynum WEI, W. Teunissen P, Varpio L. In the "Shadow of Shame": A Phenomenological Exploration of the Nature of Shame Experiences in Medical Students. Acad Med. 2021;96(11S):S23. doi:10.1097/ACM.0000000000004261	
Bynum WEI, Artino ARJ, Uijtdehaage S, Webb AMB, Varpio L. Sentinel Emotional Events: The Nature, Triggers, and Effects of Shame Experiences in Medical Residents. Acad Med. 2019;94(1):85-93. doi:10.1097/ACM.0000000000002479	
Bynum IV WE, Goodie JL. Shame, guilt, and the medical learner: ignored connections and why we should care. Med Educ. 2014;48(11):1045-1054. doi:10.1111/medu.12521	
Yilmaz Y, Carey R, Chan TM, Bandi V, Wang S, Woods RA, Mondal D, Thoma B. Developing a dashboard for faculty development in competency-based training programs: a design-based research project. Canadian Medical Education Journal/Revue canadienne de l'éducation médicale. 2021;12(4):48-64.	Assessment requires flexible systems and people who can adapt to real clinical contexts
Gingerich A, Ramlo SE, van der Vleuten CP, Eva KW, Regehr G. Inter-rater variability as mutual disagreement: identifying raters' divergent points of view. Advances in Health Sciences Education. 2017 Oct;22(4):819-38.	

Li SA, Acai A, Sherbino J, Chan TM. The teacher, the assessor, and the patient protector: a conceptual model describing how context interfaces with the supervisory roles of academic emergency physicians. AEM education and training. 2021 Jan;5(1):52-62.	
Whitehead CR, Austin Z, Hodges BD. Flower power: the armoured expert in the CanMEDS competency framework?. Advances in Health Sciences Education. 2011 Dec;16(5):681-94.	
Norman G, Norcini J, Bordage G. Competency-based education: milestones or millstones?. Journal of graduate medical education. 2014 Mar;6(1):1-6.	
Hays RB, Hamlin G, Crane L. Twelve tips for increasing the defensibility of assessment decisions. Medical Teacher. 2015 May 4;37(5):433-6.	Clinical Competency Committees and assessment systems must continually adapt—using flexible processes, evolving tools, and context-sensitive judgment—to make defensible, meaningful decisions
Acai A, Li SA, Sherbino J, Chan TM. Attending emergency physicians' perceptions of a programmatic workplace-based assessment system: the McMaster Modular Assessment Program (McMAP). Teaching and Learning in Medicine. 2019 Aug 8;31(4):434-44.	
Kinnear B, Warm EJ, Hauer KE. Twelve tips to maximize the value of a clinical competency committee in postgraduate medical education. Medical Teacher. 2018 Nov 2;40(11):1110-5.	
Ekpenyong A, Baker E, Harris I, Tekian A, Abrams R, Reddy S, Park YS. How do clinical competency committees use different sources of data to assess residents' performance on the internal medicine milestones? A mixed methods pilot study. Medical Teacher. 2017 Oct 3;39(10):1074-83.	
Chan T, Oswald A, Hauer KE, Caretta-Weyer HA, Nousiainen MT, Cheung WJ, ICBME Collaborators. Diagnosing conflict: Conflicting data, interpersonal conflict, and conflicts of interest in clinical competency committees. Medical Teacher. 2021 Jul 3;43(7):765-73.	
Thoma B, Bandi V, Carey R, Mondal D, Woods R, Martin L, Chan T. Developing a dashboard to meet Competence Committee needs: a design-based research project. Canadian Medical Education Journal. 2020 Mar;11(1):e16.	
Hauer KE, Chesluk B, Iobst W, Holmboe E, Baron RB, Boscardin CK, Ten Cate O, O'Sullivan PS. Reviewing residents' competence: a qualitative study of the role of clinical competency committees in performance assessment. Academic medicine. 2015 Aug 1;90(8):1084-92.	
Oswald A, Dubois D, Snell L, Anderson R, Karpinski J, Hall AK, Frank JR, Cheung WJ. Implementing competence committees on a national scale: design and lessons learned. Perspectives on Medical Education. 2024 Feb 6;13(1):56.	
